# Supplementary material for: The Performance of a Deep Learning-Based Automatic Measurement Model for Measuring the Cardiothoracic Ratio on Chest Radiographs
Source: Bioengineering (Basel). 2023 Sep 12;10(9):1077. doi: 10.3390/bioengineering10091077 (PMC10525628; doi:10.3390/bioengineering10091077)
Supplement: Supplementary file 1 [file bioengineering-10-01077-s001.zip › bioengineering-2513019-supplementary.pdf]

Supplementary Materials

Table S1. Mean absolute error and root mean square error for measuring the cardiothoracic ratio on chest radiographs between a deep learning-based model and two thoracic radiologists

	Session	Deep learning-based model vs. two thoracic radiologists		Deep learning-based model vs. thoracic radiologist 1		Deep learning-based model vs. thoracic radiologist 2	
		MAE	RMSE	MAE	RMSE	AME	RMSE
Study sample (n=160)	1, 2	0.019	0.028	0.021	0.030	0.017	0.026
Chest radiographs without any lung or pleural abnormality (n=40)	1, 2	0.013	0.021	0.014	0.024	0.013	0.017
Chest radiographs with pneumothorax (n=40)	1, 2	0.013	0.020	0.015	0.022	0.011	0.018
Chest radiographs with pleural effusion (n=40)	1, 2	0.025	0.033	0.028	0.034	0.023	0.031
Chest radiographs with consolidation (n=40)	1, 2	0.024	0.035	0.026	0.038	0.022	0.032
Study sample (n=160)	1	0.019	0.028	0.021	0.030	0.018	0.027
Chest radiographs without any lung or pleural abnormality (n=40)	1	0.014	0.021	0.015	0.024	0.013	0.017
Chest radiographs with pneumothorax (n=40)	1	0.012	0.018	0.013	0.019	0.012	0.018
Chest radiographs with pleural effusion (n=40)	1	0.026	0.034	0.028	0.035	0.023	0.033
Chest radiographs with consolidation (n=40)	1	0.024	0.036	0.026	0.037	0.023	0.034
Study sample (n=160)	2	0.019	0.028	0.020	0.030	0.017	0.025
Chest radiographs without any lung or pleural abnormality (n=40)	2	0.013	0.021	0.013	0.023	0.012	0.018
Chest radiographs with pneumothorax (n=40)	2	0.014	0.022	0.016	0.025	0.011	0.018
Chest radiographs with pleural effusion (n=40)	2	0.024	0.031	0.027	0.033	0.022	0.029
Chest radiographs with consolidation (n=40)	2	0.023	0.035	0.026	0.038	0.021	0.031

MAE: mean absolute error; RMSE: root mean square error

Table S2. Diagnostic performance for cardiomegaly on chest radiographs (threshold of 0.55) between a deep learning-based model and five board-certified radiologists with a reference standard derived using the Dawid-Skene consensus method

		Sensitivity	Specificity	Positive predictive value	Negative predictive value	Accuracy
Study sample	Deep learning-based model	94.5% (84.9% - 98.9%)	94.3% (88.0% - 97.9%)	89.7% (78.8% - 96.1%)	97.1% (91.6% - 99.4%)	94.4% (89.6% - 97.4%)
	Radiologists	96.7% (94.1% - 98.2%)	93.5% (89.6% - 96.0%)	88.7% (81.3% - 93.4%)	98.2% (96.5% - 99.1%)	94.6% (92.0% - 96.4%)
	P-value*	.003	<.001	.001	<.001	<.001
Chest radiographs without any lung or pleural abnormality	Deep learning-based model	100% (73.5% - 100%)	89.3% (71.8% - 97.7%)	80.0% (51.9% - 95.7%)	100% (86.3% - 100%)	92.5% (79.6% - 98.4%)
	Radiologists	96.7% (88.7% - 99.1%)	91.4% (79.3% - 96.7%)	82.9% (60.3% - 93.9%)	98.5% (93.8% - 99.6%)	93.0% (84.5% - 97.0%)
	P-value*	<.001	.046	.158	<.001	.002
Chest radiographs with pneumothorax	Deep learning-based model	100% (71.5% - 100%)	100% (88.1% - 100%)	100% (71.5% - 100%)	100% (88.1% - 100%)	100% (91.2% - 100%)
	Radiologists	96.4% (87.8% - 99.0%)	95.9% (88.3% - 98.6%)	89.8% (71.1% - 96.9%)	98.6% (94.3% - 99.7%)	96.0% (90.7% - 98.3%)
	P-value*	<.001	<.001	<.001	<.001	<.001
Chest radiographs with pleural effusion	Deep learning-based model	93.3% (68.1% - 99.8%)	96.0% (79.6% - 99.9%)	93.3% (68.1% - 99.8%)	96.0% (79.6% - 99.9%)	95.0% (83.1% - 99.4%)
	Radiologists	97.3% (90.7% - 99.3%)	90.4% (80.7% - 95.5%)	85.9% (69.6% - 94.2%)	98.3% (93.0% - 99.6%)	93.0% (86.6% - 96.5%)
	P-value*	.191	<.001	.004	.028	.001
Chest radiographs with consolidation	Deep learning-based model	88.2% (63.6% - 98.5%)	91.3% (72.0% - 98.9%)	88.2% (63.6% - 98.5%)	91.3% (72.0% - 98.9%)	90.0% (76.3% - 97.2%)
	Radiologists	96.5% (90.4% - 98.8%)	96.5% (89.6% - 98.9%)	95.3% (84.9% - 98.7%)	97.4% (91.7% - 99.2%)	96.5% (92.5% - 98.4%)
	P-value*	.392	.228	.359	.199	.224

* One-sided non-inferiority test with a cut-off of a *P* value of .025

Table S3. Diagnostic performance for cardiomegaly on chest radiographs between a deep learning-based model and five board-certified radiologists with a reference standard derived using the median values

		Sensitivity	Specificity	Positive predictive value	Negative predictive value	Accuracy
Study sample	Deep learning-based model	97.6% (91.7% - 99.7%)	86.8% (77.1% - 93.5%)	89.1% (80.9% - 94.7%)	97.1% (89.8% - 99.6%)	92.5% (87.3% - 96.1%)
	Radiologists	97.9% (95.8% - 98.9%)	87.4% (81.9% - 91.4%)	89.5% (84% - 93.3%)	97.4% (94.5% - 98.7%)	92.9% (89.9% - 95%)
	P-value*	<.001	.002	<.001	<.001	<.001
Chest radiographs without any lung or pleural abnormality	Deep learning-based model	100% (83.9% - 100%)	94.7% (74% - 99.9%)	95.5% (77.2% - 99.9%)	100% (81.5% - 100%)	97.5% (86.8% - 99.9%)
	Radiologists	100% (96.5% - 100%)	95.8% (87.7% - 98.6%)	96.3% (88% - 98.9%)	100% (96% - 100%)	98% (93.8% - 99.4%)
	P-value*	-	.032	.011	-	<.001
Chest radiographs with pneumothorax	Deep learning-based model	100% (81.5% - 100%)	86.4% (65.1% - 97.1%)	85.7% (63.7% - 97%)	100% (82.4% - 100%)	92.5% (79.6% - 98.4%)
	Radiologists	96.7% (87.4% - 99.2%)	89.1% (78.4% - 94.8%)	87.9% (73.6% - 95%)	97% (87.7% - 99.3%)	92.5% (85.8% - 96.2%)
	P-value*	<.001	.074	.04	<.001	<.001
Chest radiographs with pleural effusion	Deep learning-based model	95.7% (78.1% - 99.9%)	76.5% (50.1% - 93.2%)	84.6% (65.1% - 95.6%)	92.9% (66.1% - 99.8%)	87.5% (73.2% - 95.8%)
	Radiologists	95.7% (90.7% - 98%)	81.2% (67.7% - 89.9%)	87.3% (74.4% - 94.2%)	93.2% (83.5% - 97.4%)	89.5% (82.5% - 93.9%)
	P-value*	.005	.276	.072	.054	.035
Chest radiographs with consolidation	Deep learning-based model	95.5% (77.2% - 99.9%)	88.9% (65.3% - 98.6%)	91.3% (72% - 98.9%)	94.1% (71.3% - 99.9%)	92.5% (79.6% - 98.4%)
	Radiologists	99.1% (94% - 99.9%)	82.2% (67.2% - 91.2%)	87.2% (73.2% - 94.4%)	98.7% (90.8% - 99.8%)	91.5% (83.2% - 95.9%)
	P-value*	.082	.01	.003	.173	.004

* One-sided non-inferiority test with a cut-off of a *P* value of .025; Statistical comparisons and confidence intervals for the difference between the diagnostic measures cannot be estimated in case the deep learning-based model and radiologists have 100% of the sensitivity and negative predictive values.

Figure S1. Plots of cardiothoracic ratio measurements of radiologists (X-axis) and deep learning-based model (Y-axis). (A-E) Reference standard for cardiomegaly constructed by Dawid-Skene consensus method: (A) study sample, (B) chest radiographs without any lung or pleural abnormality, (C) chest radiographs with pneumothorax, (D) chest radiographs with pleural effusion, and (E) chest radiographs with consolidation. (F-J) Reference standard for cardiomegaly constructed by median values: (F) study sample, (G) chest radiographs without any lung or pleural abnormality, (H) chest radiographs with pneumothorax, (I) chest radiographs with pleural effusion, and (J) chest radiographs with consolidation. Green solid circle: cardiomegaly by both radiologists and a deep learning-based model; Orange solid circle: cardiomegaly only by a deep learning-based model; Orange open circle: cardiomegaly only by radiologists; Green open circle: normal by both radiologists and deep learning-based model

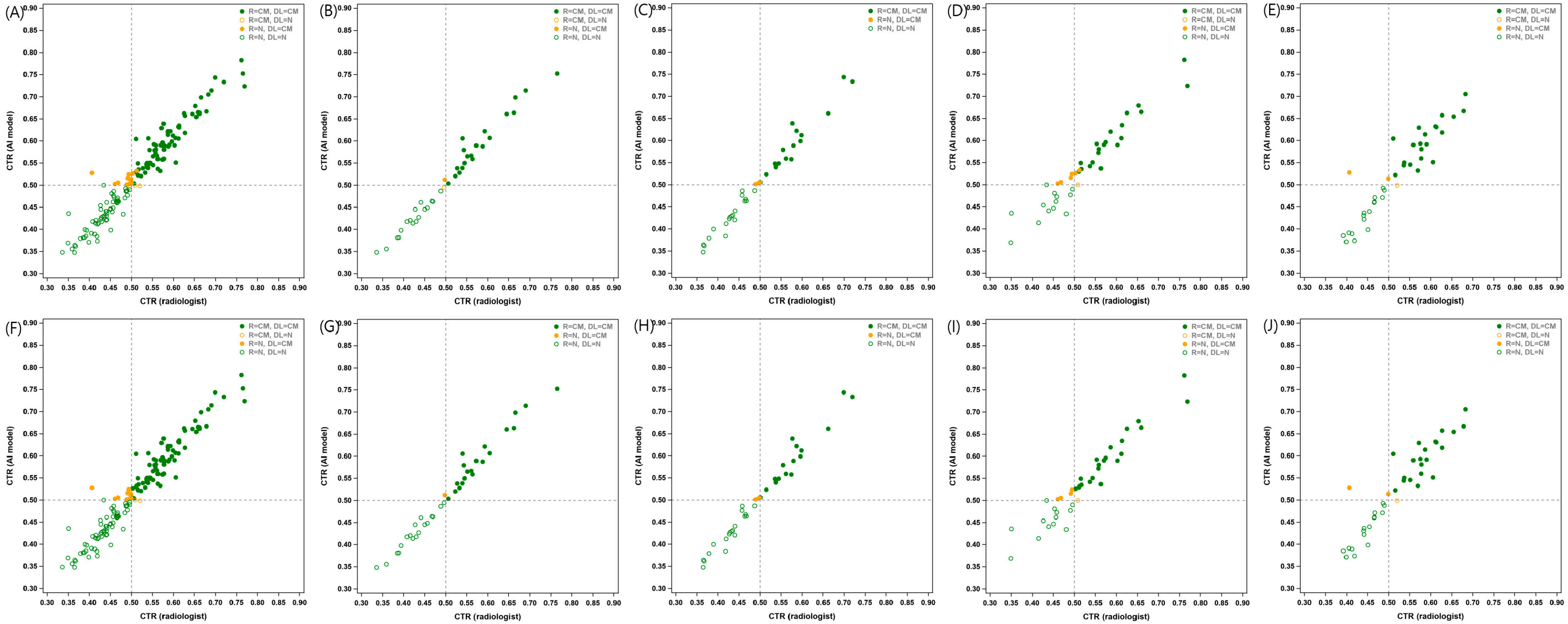

CTR: cardiothoracic ratio; R: radiologists; DL: deep learning; CM: cardiomegaly
